# Utilization of Pre-Anesthetic Medications for Major Surgical Procedures at a Tertiary Care Center: A Descriptive Cross-sectional Study

**DOI:** 10.31729/jnma.4841

**Published:** 2020-04-30

**Authors:** Rekha Shah, Roshan Pradhan, Arbindra Shah

**Affiliations:** 1 Department of Pharmacology, Birat Medical College, Biratnagar, Nepal; 2 Department of Anesthesiology, Birat Medical College, Biratnagar, Nepal; 3 Department of Radiology, Nobel Medical College, Biratnagar, Nepal

**Keywords:** *pre-anesthetic medications*, *surgical procedures*, *utilization*

## Abstract

**Introduction:**

Drug utilization research is an important tool to analyze the use of drugs with special emphasis on medical, social, and economic consequences in society. This study aims to find out the utilization of pre-anesthetic medications in a major surgical procedure.

**Methods:**

A descriptive cross-sectional study was conducted from 15th April - 15th August 2019 in the postoperative ward at Birat Medical College and Teaching Hospital. The convenience sampling method was used after ethical clearance from the Institutional Review Committee (IRC) of Birat Medical College and Teaching Hospital, Biratnagar, Nepal. About 400 patients were studied. The collected data were entered into a statistical package for social science version 20 for further calculations at 95% Confidence Interval.

**Results:**

Out of 400 patients, 215 (53.8%) of patients were underwent into different major surgeries. All patients received midazolam 2 mg except children (1 mg) and Pethidine 25 mg along with 0.2 mg glycopyrrolate 352 (88%), ondansetron 276 (69%) and others 58 (14.5%) as a preanesthetic agent. For general anesthesia propofol, 30 mg have been utilized followed by fentanyl 306 (76.5%) and others (halothane, isoflurane, etc) 115 (28.8%). In case of prophylactic drug were ceftriaxone 500 mg, 100 mg metoclopramide 387 (96.8%), dexamethasone 251 (62.8%), tramadol 237 (59.3%), 15 mg ketorolac 368 (92%), ranitidine 163 (40.8%), and pantoprazole 237 (59.3%).

**Conclusions:**

The most commonly administered pre-anesthetic drugs were midazolam, pethidine, glycopyrrolate, and ondansetron. The incidence of postoperative nausea and vomiting the patient within 24 hours after surgery was significantly very low.

## INTRODUCTION

Every year worldwide, approximately more than 310 million operations are performed,^1^ among them more than 200 million patients underwent major surgery.^2^ Pre-anesthetic medications are the drugs used before administration of an anesthetic agent.^3^ Introduction of such medication play the key role for improvement in the quality of health by counteracting stress and fear of surgery.^4,5^

About one-third of surgical patients received general anesthesia experience postoperative nausea and vomiting (PONV).^6^ After pain, PONV is the second most complaint in the post-operative ward. The patients who are not under PONV prophylaxis, the incidence rate of PONV was 70-80 %.^7^ To minimized the PONV several new drugs have been introduced but the incidence rate remains significantly high, ranging from 30%-35%.^8^ So to reduce it or to improve and for better prophylaxis combinations of drugs are used.^9—14^

As per our knowledge, this type of study has not been done yet in such settings so this study was designed to find out the utilization of pre-anesthetic medications in a major surgical procedure.

## METHODS

This descriptive cross-sectional study was conducted from 15th April to 15th October 2019 at Birat Medical College and Teaching Hospital, Biratnagar, Nepal. Ethical clearance was taken from the Institutional Review Committee (IRC) of Birat Medical College and Teaching Hospital, Biratnagar, Nepal. Written permission was taken from each participant of the study. Inclusion criteria were those who gave consent or all patient of the postoperative ward who was willing to participate in the study and the participants of both sexes aged 6 years to 75 years. Exclusion criteria were those that do not give consent, age below 6 years and above 75 years. A semi-structured questionnaire and an observational checklist were used for data collection and face to face interviews were taken. The confidentiality and privacy of the study were maintained; the name of the individuals or the participating group was not disclosed after the study.

For the data collection, the pre-structured questionnaire was made by reviewing different literature and seeking opinions with the experts on the subject than appropriate modification was done. All patients aged from 6 to 70 years operated under general anesthesia given different pre-anesthetic medication before major surgery was noted in the questionnaire from the relevant patient file record. Data was collected two times 1st before operation from the patient's with present record file then after operation by face-to-face interview at the bedside along with record file in the post-operative ward. The confidentiality and privacy of the study were maintained; the name of the individuals or the participating group was not disclosed after the study. Here in this study, the side effect most commonly was seen was nausea and vomiting which is also called postoperative nausea and vomiting (PONV), therefore these have been noted.

Convenience sampling was done. The sample size was calculated as follow,

$$\begin{array}{ll} \text{n} & \text{= }{\text{Z}}^{\text{2}}\times \text{p }\times \text{q}/{\text{e}}^{\text{2}}\\ & \text{= 3.84}\times 0.5\times 0.5/{\text{(0.05)}}^{\text{2}}\\ & \text{= }384 \end{array}$$

where,

n = minimum sample size
p = prevalence, 50%
q = 1-p
e = margin of error, 5%
Z = 1.96 at 95 % CI

After taking a non-response rate of 4%, the total calculated sample for the study was taken to be 400 cases. Different biases present in the study such as selection bias and interviewer bias has been minimized as much possible. The collected data was entered in Microsoft excel and converted into SPSS (Statistical Package for Social Science) software package 20 version for statistical analysis. Data were analyzed using descriptive statistics for drug utilization. Point estimate at 95% CI was done along with frequency and proportion for binary data.

## RESULTS

Laparoscopic appendectomy 124 (31%) becomes the highly done procedure followed by lab cholecystectomy 121 (30.3%) (Table 1). Midazolam 2 mg and pethidine 20 mg were utilized to all patients as a pre-anesthetic medication. The drugs administered as post-operative prophylaxis were ceftriaxone 500 mg to all patients followed by 100 mg metoclopramide.

**Table 1:** Utilization of medications in major surgery.

Characteristics	n (%)
Procedure	
Lab appendectomy	124 (31.0)
Laparoscopic cholecystectomy	121 (30.3)
Cortical mastoidectomy	23 (5.8)
Left tympanoplasty	41 (10.3)
Tonsillectomy	35 (8.8)
Excision of fibroadenoma	12 (3.0)
Open cholecystectomy	21 (5.3)
Others (reduction, perforation Peritonitis, etc)	23 (5.8)
Pre-anesthetic medication	
Glycopyrrolate	
Yes	352 (88.0)
No	48 (12.0)
Ondansetron	
Yes	276 (69.0)
No	124 (31.0)
Others (buscopan, ketorolac, etc)	
Yes	58 (14.5)
No	342 (85.5)
General Anesthetics	
Fentanyl	
Yes	306 (76.5)
No	94 (23.5)
Others (halothane, isoflurane, etc)	
Yes	115 (28.8)
No	285 (71.3)
Post-operative drugs	
Metoclopramide	
Yes	387 (96.8)
No	13 (3.3)
Dexamethasone	
Yes	251 (62.8)
No	149 (37.3)
Tramadol	
Yes	237 (59.3)
No	163 (40.8)
Ketorolac	
Yes	368 (92.0)
No	32 (8.0)
Ranitidine	
Yes	163 (40.8)
No	237 (59.3)
Pantoprazole	
Yes	237 (59.3)
No	162 (40.5)

Among the side effects nausea and vomiting being the most highly seen under post-operative drugs and pre-anesthetic medication in the post-operative ward (Table 2).

**Table 2:** Side effects observed under post-operative drugs and pre-anesthetic medication patients.

Characteristics	n (%)
Side effects	
Yes	119 (29.8)
No	281 (70.3)
Nausea	
Yes	84 (21.0)
No	316 (79.0)
Vomiting	
Yes	73 (18.3)
No	
Others (pruritus, abdominal cramp, headache)	327 (81.8)
Yes	37 (9.3)
No	363 (90.8)
Total	400 (100)

Females were showed slightly higher side effects 75 (30.4%) as compared to males 44 (28.8%) but the difference was not significant. The relation between common Side effects (PONV) with Pre-anesthetic medication along with post-operative prophylaxis drugs was highly significant (Table 3).

**Table 3:** Association between age, gender, pre-anesthetic medication, and prophylaxis drugs with postoperative common side effects (PONV).

Characteristics	Side effects within 24hrs after surgery		Total n (%)
	Yes n (%)	No n (%)	
Age			
<18 years	6 (22.2)	21 (77.8)	27 (100)
18-24 years	25 (36.8)	43 (63.2)	68 (100)
25-39 years	63 (29.3)	152 (70.7)	215 (100)
40-59 years	21 (28.0)	54(72.0)	75 (100)
60 years	4 (26.7)	11(73.3)	15 (100)
Gender			
Male	44 (28.8)	109 (71.2)	153 (100)
Female	75 (30.4)	172 (69.6)	247 (100)
**Pre-anesthetic medication and prophylaxis drugs.**			
M, D, G, O^*^	15 (13.3)	98 (86.7)	113 (100)
M, D, G^†^	11 (19.0)	47 (81.0)	58 (100)
D, G, O^‡^	39 (39.0)	61 (61.0)	100 (100)
M, G^§^	13 (65.0)	7 (35.0)	20 (100)
M, G, O^||^	23 (46.0)	27 (54.0)	50 (100)
M, O^¶^	12 (30.0)	28 (70.0)	40 (100)
M, D, O^**^	6 (31.6)	13 (68.4)	19 (100)
Total	119 (29.8)	281 (70.3)	400 (100)

M, D, G, O* = metroclopramide, dexamethasone, glycopyrolate, ondansetrone

M, D, G† = metroclopramide, dexamethasone, glycopyrolate,

D, G, O‡ = dexamethasone, glycopyrolate, odensetrone

M, G§ = metroclopramide, glycopyrolate

M, G, O|| = metroclopramide, glycopyrolate, odensetrone

M, O¶ = metroclopramide, odensetrone

M, D, O** = metroclopramide, dexamethasone, odensetrone

The majority of the study population i.e. 215 (53.8%) belong to age groups of 25-39 years and minority i.e. 15 (3.8%) belong to the age group above 60 years. Almost 153 (38.3%) of the male were dominated by female 247 (61.8%) for the utilization of pre-anesthetic medication (Table 4).

**Table 4:** Distribution of study population by age and gender.

Characteristics	n (%)
Age	
<18 years	27 (6.8)
18-24 years	68 (17.0)
25-39 years	215 (53.8)
40-59 years	75 (18.8)
60 years	15 (3.8)
Gender	
Male	153 (38.3)
Female	247 (61.8)
Total	400 (100)

## DISCUSSION

Globally, 234.1 million major surgical procedures have been carried out every year.^2^ For major surgery, propofol from general anesthesia was mostly utilized in Di Filippo, et al,^15,16^ which was similar to our finding. Propofol was used for induction and maintenance purposes. This has a peculiar feature associated with earlier discharge from the post-anesthesia care unit.^17,18^ General anesthesia and surgery were responsible to develop postoperative complication like Postoperative Nausea and Vomiting (PONV)^19,20^ although there are multi-factorial reasons for PONV.^21–24^ So to compensate for the above postoperative complication appropriate drugs in Preanesthetic medicine and prophylaxis drugs were utilized before and after surgery to reduce the adverse effect.

In our study identical premedication and standardized anesthetic technique^25^ (Balanced general anesthesia) was applied to all the patients who underwent major surgery. There were different surgical procedure estimated in this study, the major 3 operations which were highly done were laparoscopic (lap) appendectomy, 31.0% followed by laparoscopic (lap) cholecystectomy, 30.3%, Lf Tympanoplasty 10.3%, whereas, in the retrospective study conducted by Biondi A, et al.^26^ in 2016, 283 patients treated by laparoscopic appendectomy out of 593, Bajracharya A, et al.^27^ showed 346 laparoscopic cholecystectomies done at 2010 where the female was dominant on undergoing surgery. In our study, almost 61.8% of the female have dominated male patients 38.3%. Among 400 patients in our study, age group of 25-39 years has undergone surgery in the high category about 53.8% similarly a retrospective study conducted on 2019 by Gajjala R, et al. in which maximum surgeries were carried at the adult's age group 25-39 years, 49%.28 Santos ML, et al.^29^ did study on 2017, found similar age group of patients 18-59 years, 55.9%. This fact expresses that there is a progressive increase in surgery in younger age population. This implies the need for trained health professionals and institutions to care for this growing group of patients.

Among the pre-anesthetic medication, the benzodiazepine group of the drug was highly prescribed drugs.^30^ In our study Midazolam was commonly utilized drugs similar to finding Sheen MJ, et al.^31^ study whereas these findings differ from the studies of Patil A,^32^ Biswas P, et al.^30^ where the most commonly used anxiolytic was alprazolam. Here the glycopyrrolate 352 (88%) has been utilized which were more potent than atropine, used routinely as a pre-medication purpose and have less risk to the cardiovascular system, better control of oropharyngeal secretions during reversal stage^8^ when compared to Zeev N and Mirakur RK, et al. where atropine was the most commonly used.^33,34^ Another most important drugs utilized in our study as a pre-anesthetic medication was Ondansetron (69.0%) from antiemetic drugs. Establishment of 5-HT3 receptor antagonists approved by FDA brings drastic change in the prevention of postoperative nausea and vomiting.^35^

In this study there were a different type of major surgery has been done under general anesthesia where there was a high risk of incidence of PONV, especially observed in female gender similar to many studies done by Apfel, et al.^36^ Sinclair, et al.^37^ and Koivuranta, et al.^38^ found, to compensate the PONV different prophylactic drugs along with pre-anesthetic medication have been administered. Because the single drug was not 100% effective so the concept of combination antiemetic therapy was utilized which was first introduced in chemotherapy-induced vomiting.^39^ Several other studies in the literature also demonstrated that combined use of dexamethasone with other antiemetics decreases the incidence of PONV.^40,41^ Similar effectiveness was obtained in our study by the patients under prophylactic dexamethasone and metoclopramide along with administration of glycopyrrolate and ondansetron as pre-anesthetic medication showed very less side effect i.e. 15 (13.3%) out of 113 followed by metoclopramide, dexamethasone while glycopyrrolate has 11 (19.0%) out of 58 followed by metoclopramide, dexamethasone while ondansetron has 6 (31.6%) out of 19 respectively. This showed that prophylactic drugs along with pre-anesthetic medication were highly significant with side effects.

In our study population not only prophylaxis anti-emetic have been utilized but also other drugs such as pantoprazole, ranitidine, ketorolac, tramadol and antibiotic to compensate the side effect produce during and after surgery. Pantoprazole was the most highly utilized drugs followed by ranitidine in our study; the finding was similar to a study by Biswas P, et al.^30^ done in 2014. In a meta-analysis by Clark K, et al. found the opposite result where H2 blockers were effective drug compared to proton pump inhibitor (PPI) in reducing the gastric volume and pH.^42^

Surgery is one of the painful procedures so to reduce, strong analgesic was utilized here such as ketorolac 368 (92.0%), tramadol 237 (59.3%) and short-acting opioid, fentanyl 306 (76.5%). Fentanyl being the strong pain reliever but decrease the hemodynamic response to tracheal intubation in high dose.^43^ For these reasons, non-opioid agents to opioid-based regimens were added. Ketorolac is a common adjunct in major surgical protocols.^44^ According to the plethora of guidelines and study reporting the use of third-generation cephalosporins, ceftriaxone for surgical prophylaxis which was also followed in our study.^45^

In this study only the most commonly observed side effect that is nausea and vomiting within 24 hours of operation have been recorded but late PONV, as well as another individual side effect, was not able to measure. We were not able to record all the PONV promptly within 24 hours after surgery, which has undergone major surgery. Therefore, there may be a chance of observational bias done by nursing staff during recording side effects.

## CONCLUSIONS

The most commonly administered Pre-anesthetic drugs are midazolam followed by pethidine was highly utilized pre-anesthetic medication. The other Preanesthetic medication were less utilized compared to above were glycopyrrolate, ondansetron, and others (buscopan, ketorolac, etc). The incidence of PONV to the patient within 24 hours after surgery was significantly very low to the patients who received glycopyrrolate and ondansetron as Preanesthetic medication along with metoclopramide and dexamethasone (MDGO) as prophylaxis drugs compared to another group of drugs in postoperative ward.
